# Astrocyte lipid metabolism in brain health and neurodegeneration: from regulatory hub to maladaptive stressor

**DOI:** 10.1186/s13024-026-00981-x

**Published:** 2026-08-01

**Authors:** Weihua Wang, Huajun Pan, Yashi Mi, Juvie Farol, Fei Yin

**Affiliations:** 1 Center for Innovation in Brain Science, University of Arizona, 1230 N Cherry Ave, BSRL 471, Tucson, AZ 85721 USA; 2 Department of Pharmacology, College of Medicine Tucson, University of Arizona, Tucson, AZ USA; 3 Graduate Interdisciplinary Program in Neuroscience, University of Arizona, Tucson, AZ USA

**Keywords:** Astrocyte, Neurodegenerative diseases, Cholesterol, Fatty acids, APOE, Lipid droplets

## Abstract

Neurodegenerative diseases are increasingly recognized as multicellular pathologies in which astrocytes serve as active determinants of disease onset and progression. Central to the regulatory role of astrocytes is the precise and dynamic control of brain lipid homeostasis. This review discusses a framework positioning astrocytic lipid metabolism as a key axis of both brain metabolic health and neurodegenerative processes. Under physiological conditions, astrocytes operate as the primary lipid metabolic hub, coordinating *de novo* cholesterol synthesis, APOE-mediated lipid transport, and the uptake and degradation of fatty acids. However, upon aging, chronic stress, and proteinopathies, these homeostatic programs undergo profound adaptation and remodeling. We detail how the breakdown of astrocytic lipid handling—driven by genetic risk factors such as APOE4 and manifesting as cholesterol dyshomeostasis, aberrant lipid droplet accumulation, impaired lipid turnover, and pro-inflammatory lipid signaling—transforms astrocytes into maladaptive reactive states and contributes to neurodegeneration. This metabolic rewiring not only deprives vulnerable neurons of essential metabolic and structural support but actively amplifies neuroinflammation and neurotoxicity. Finally, we consider therapeutic strategies aimed at restoring functional lipid flux and mitigating lipid-driven pathogenic signaling. By examining neurodegeneration through the lens of astrocytic lipid metabolism, we highlight novel conceptual paradigms for understanding neurodegeneration and emerging translational opportunities for disease interventions.

## Introduction

Neurodegenerative diseases are a heterogeneous group of central nervous system (CNS) disorders characterized by progressive loss of neuronal function and integrity, yet they present with diverse clinical manifestations and pathological hallmarks [1]. While these disorders have been extensively examined through neuron-centered approaches, with emphasis on protein aggregation, selective neuronal vulnerability, progressive synaptic failure, and ultimately neuronal death, it is now clear that neurodegeneration involves all major brain cell types and unfolds within complex multicellular environments. Astrocytes, in particular, are increasingly recognized as active regulators of both tissue homeostasis and disease progression. Under physiological conditions, astrocytes support a variety of essential brain functions, including maintenance of neurotransmitter and ion homeostasis, antioxidant defense, modulation of synapse function, and support of neurovascular coupling and blood-brain barrier (BBB) integrity (reviewed in [2–7]). In response to aging, proteotoxic stress, or chronic inflammation, these homeostatic functions are remodeled into reactive states that may either limit or exacerbate neuronal damage [8–11]. Astrocytes should therefore be viewed not only as secondary responders, but also as integral modulators of neurodegenerative pathobiology.

Among astrocyte-regulated processes implicated in brain health and disease, lipid metabolism is of particular importance because the brain maintains a highly specialized and relatively autonomous lipid environment. Circulating lipoprotein cholesterol does not substantially cross the BBB, and brain cholesterol therefore depends largely on local synthesis and intracerebral trafficking [12–14]. At the same time, selected fatty acid (FA)-derived lipids can enter the brain through regulated transport systems [15], suggesting that lipid exchange across the BBB is restricted and selective. Within this specialized environment, astrocytes are the principal source of *de novo* cholesterol and APOE-containing lipoproteins for surrounding cells, and thereby influence membrane biogenesis, synaptic function, energy balance, and inflammatory signaling [16–19]. Astrocyte-derived lipids also make major contributions to CNS myelination [20, 21], which extends the relevance of astrocytic lipid metabolism beyond gray matter synapses to white matter integrity, and demyelinating diseases.

Astrocytes also serve as an important buffer against lipid-related stress. Neurons can offload excess or damaged FAs to astrocytes, which either sequester them in lipid droplets (LDs) or direct them toward mitochondrial oxidative degradation [22–27]. While such metabolic coupling protects neurons from lipotoxicity, it also renders astrocytes particularly sensitive to disturbances in lipid trafficking, storage, and catabolism. In disease settings, these normally protective and adaptive pathways can become chronically rewired, leading to defective cholesterol handling, abnormal APOE lipidation, impaired lipid waste disposal and replenishment, as well as lipid-linked inflammatory signaling that collectively contribute to neurodegeneration.

In this review, we examine astrocytic lipid metabolism as a common framework for understanding neurodegenerative processes. We first present lipid metabolism within the broader context of astrocyte biology and reactive state heterogeneity. We then discuss the core physiological pathways of cholesterol synthesis, APOE lipidation, FA trafficking, LD storage, mitochondrial β-oxidation, and myelin lipid support. Further, we consider how these pathways are reshaped across disease contexts and discuss the therapeutic and conceptual opportunities that emerge when astrocytes are viewed as the homeostatic lipid regulator in brain health, and as maladaptive stress initiators or amplifiers in neurodegeneration.

## Astrocyte reactivity and metabolic vulnerability: a context for lipid metabolism

As the most abundant glial cells in the brain, astrocytes are increasingly recognized as active determinants of neurodegeneration rather than passive responders to neuronal damage or death. Their functions extend well beyond structural or trophic support and include regulation of extracellular ion and water balance, neurotransmitter clearance and recycling, antioxidant and metabolic support, synapse formation and maintenance, and neurovascular coupling and BBB integrity [2–7, 28–31]. Through these processes, astrocytes serve as hubs of multicellular communication by exchanging signals and metabolites with neurons, microglia, oligodendrocytes, endothelial cells, and pericytes. In doing so, they coordinate local adaptation to synaptic activity, metabolic demand, inflammatory tone, and vascular status. When these homeostatic programs fail, however, astrocytes can also propagate chronic stress and damage signals across cell types. Astrocytes are therefore well positioned to shape both the resilience and vulnerability of neurons and neural circuits to neurodegenerative cues [32, 33].

Astrocytes also provide essential bioenergetic and redox support to neurons, which have limited energy reserves and restricted metabolic flexibility [31]. Through the astrocyte-neuron lactate shuttle (ANLS), astrocytes convert blood- or glycogen-derived glucose into lactate to support neuronal oxidative metabolism during periods of increased activity [34]. Neurons also depend on astrocytes for antioxidant protection, including the provision of glutathione precursors and the buffering of oxidative stress (reviewed in [35]). In addition, astrocytes participate in reactive oxygen species (ROS) detoxification indirectly by accepting and degrading peroxidized lipids and other potentially toxic metabolites generated by stressed neurons (discussed in Section "Neuron-to-astrocyte FA transport, storage, and degradation"). This division of metabolic specialties reflects intrinsic neuronal constraints, including limited fuel storage, low glycolytic adaptability, restricted FA β-oxidation, and limited antioxidant capacity, all of which increase neuronal dependence on astrocytic metabolic support and buffering.

A defining disease-related adaptation of astrocytes is their reactive transformation. Astrocytes undergo molecular, morphological, and functional remodeling in response not only to injury and acute inflammation, but also to aging, genetic risk factors, and environmental perturbations, towards reactive states with distinct molecular signatures [9]. Over the past decade, the concept of astrocyte reactivity has shifted from a largely uniform response to injury toward a spectrum of context-dependent states shaped by the type of insult, disease stage, anatomical region, and local cellular environment. Accordingly, early binary views of reactive astrocyte states have evolved to a broader view of multidimensional astrocyte heterogeneity that more accurately reflects the diversity of astrocyte responses observed across neurological conditions in vivo [9, 36, 37]. Consistent with this notion, large-scale single-nucleus RNA sequencing (snRNA-seq) studies of the human brain have identified multiple transcriptionally distinct astrocyte populations associated with regional specialization, aging, and neurodegenerative disease states, further highlighting the complexity of human astrocyte heterogeneity [38–42].

This framework is equally relevant for a lipid-centered view of astrocytes in neurodegeneration. Astrocyte metabolic states are unlikely to map onto a simple neurotoxic versus neuroprotective paradigm. Instead, alterations in cholesterol synthesis, APOE lipidation, FA handling, and lipid-driven signaling are more likely to represent interacting components of broader state-dependent adaptive and maladaptive programs. In that regard, altered lipid metabolism is not merely a consequence or manifestation of astrocyte reactivity, but could be one of the mechanisms through which heterogenous reactive states are triggered, executed, and sustained.

While this review focuses on astrocytic lipid metabolism, other CNS cell types, particularly microglia, also play important roles in lipid sensing, phagocytic lipid clearance, cholesterol handling, and inflammatory lipid signaling during neurodegeneration. Similar to astrocytes, microglia accumulate LDs in response to inflammation, oxidative stress, and phagocytic burden, and excessive LD accumulation is increasingly recognized as a hallmark of activated or dysfunctional microglial states [43, 44]. LD-accumulating microglia exhibit altered transcriptional programs characterized by increased expression of lipid metabolism and lipid-sensing genes including APOE, together with impaired phagocytosis and pro-inflammatory transformation [45–47]. In particular, TREM2 functions as a key lipid sensor regulating microglial cholesterol metabolism, myelin lipid clearance, and survival, whereas disruption of TREM2 signaling promotes cholesteryl ester accumulation, defective phagocytosis, and neurodegenerative phenotype [48]. For in-depth discussions on microglial lipid metabolism and its implications in neurodegenerative diseases, we refer readers to several recent comprehensive reviews on this topic [16, 49–52].

Overall, astrocytes are state-dependent regulators of tissue homeostasis whose functions are closely linked to metabolic state and intercellular communications. Under disease conditions, their importance lies in both their own reactive transformation and how that transformation alters their ability to support neurons, communicate with other glial cells, and adapt to chronic stress. Within this context, lipid metabolism is especially relevant because it sits at the interface of these homeostatic and reactive functions, as discussed in the following sections.

## Astrocytic lipid programs maintain brain homeostasis

This section summarizes the major homeostatic pathways of astrocytic lipid metabolism, including cholesterol synthesis and trafficking, APOE lipidation, FA degradation, LD dynamics, as well as lipid metabolic coupling with neurons and myelinating glia. These physiological functions provide the baseline for understanding how astrocytic lipid pathways are implicated in neurodegeneration.

### Cholesterol synthesis, APOE lipidation, and efflux

**Fig. 1:**
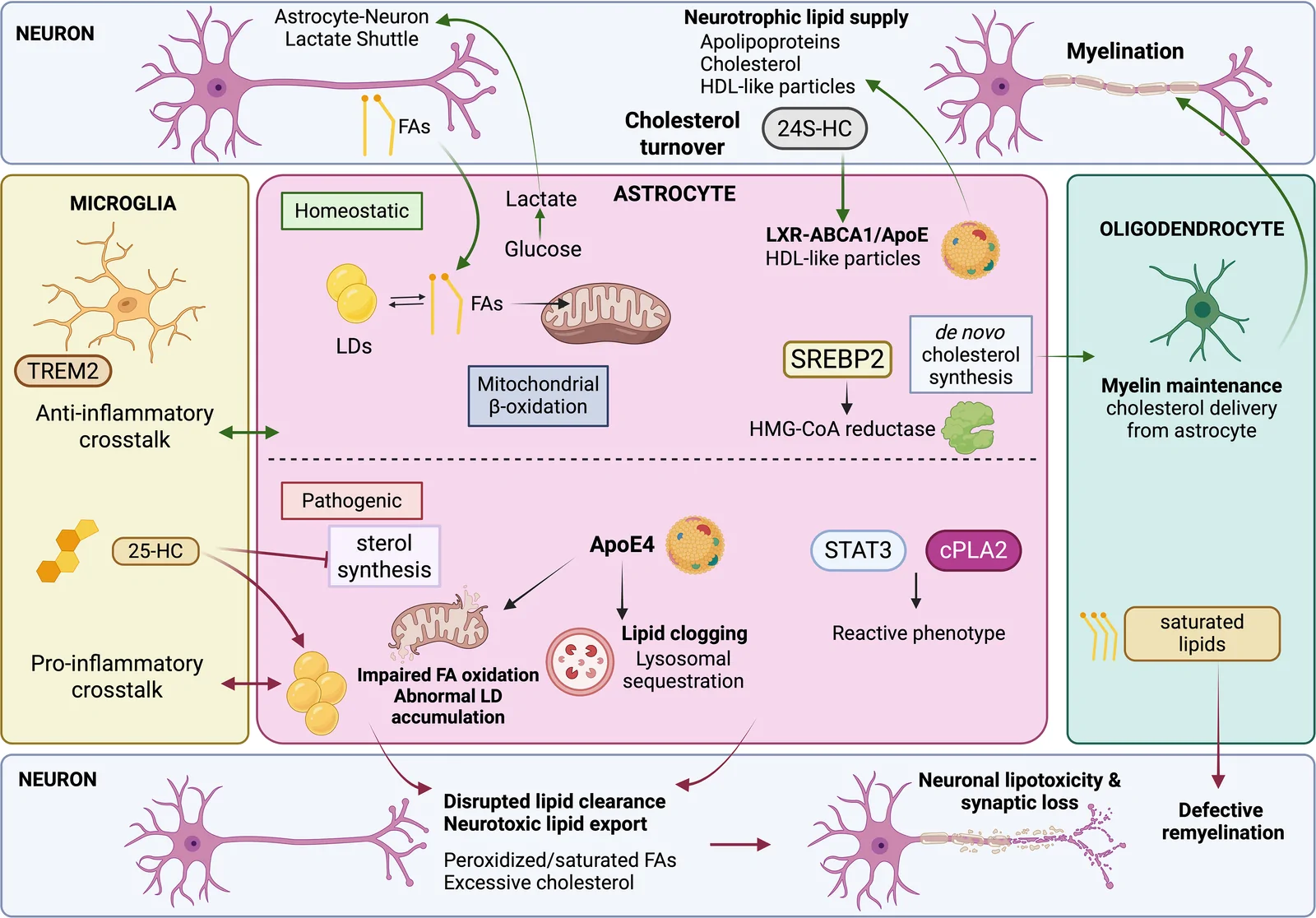
Astrocytic lipid metabolism and intercellular crosstalk. Under homeostatic conditions (top side), SREBP2 controls de novo cholesterol synthesis via HMG-CoA reductase to supply apolipoproteins and cholesterol as HDL-like particles via LXR-ABCA1/APOE pathways to support neuronal membrane integrity and synaptic stability, and myelin maintenance by oligodendrocytes. Astrocytes also support neuronal energy balance by converting glucose into lactate for the Astrocyte-Neuron Lactate Shuttle (ANLS). Astrocytes can take up excess FAs by sequestering into lipid droplets (LDs) or directing them to mitochondrial β-oxidation. Microglia participate in protective anti-inflammatory crosstalk, partially mediated by TREM2. In pathogenic conditions (bottom side), microglia-derived 25-HC suppresses sterol synthesis, and promotes abnormal peroxidized lipid droplet (LD) accumulation, contributing to a pro-inflammatory state. APOE4 induces lysosomal sequestration and lipid clogging. Lipid changes drive astrocytes transition to a reactive phenotype primarily via STAT3- and cPLA2-mediated pathways. This pathological shift also involves disrupted lipid clearance by astrocytes, impaired FA oxidation by astrocytic mitochondria, export of peroxidized and/or saturated FAs, and excessive cholesterol to neurons, which collectively contribute to neuronal lipotoxicity, synaptic loss, and defective myelin maintenance. Green arrows indicate physiological/homeostatic processes; red arrows indicate pathogenic processes

Cholesterol is an essential structural component of cellular membranes and a key determinant of membrane nanodomain organization, including lipid rafts that support signal transduction and membrane trafficking [53–56]. In the brain, cholesterol homeostasis is regulated independently of peripheral tissues because circulating lipoprotein cholesterol does not substantially cross the BBB. Neurons therefore rely predominantly on local sterol synthesis and intracerebral trafficking. Within the brain, astrocytes are the principal net producers and distributors of cholesterol, placing them as the hub of CNS cholesterol metabolism. In astrocytes, cholesterol synthesis is controlled by sterol-sensitive transcriptional programs centered on sterol regulatory element-binding protein 2 (SREBP2) (Fig. 1), which induces the expression of key biosynthetic enzymes including the rate-limiting HMG-CoA reductase [57–59].

Newly synthesized cholesterol is then mobilized for export, which links intracellular synthesis directly to intercellular lipid delivery. Apolipoprotein E (APOE) is the major apolipoprotein of CNS lipoprotein particles, which mediate the transport and redistribution of cholesterol and phospholipids across cells or organs. In the CNS, APOE-mediated lipid transport is required for synaptic maintenance and neuronal homeostasis, with APOE predominantly produced by astrocytes under physiological conditions [59–62]. Other apolipoproteins, including APOJ/clusterin (CLU), APOA-I, and APOD, also contribute to brain lipid biology and have been implicated in AD-related lipid dysregulation, amyloid processing, and neuroinflammatory or stress responses, although APOE remains the dominant apolipoprotein mediator of lipid transport in the adult CNS [63–67]. Astrocyte-derived cholesterol is exported on APOE-containing lipoproteins in a tightly regulated process that depends primarily on ATP-binding cassette (ABC) transporters, specifically ABCA1-mediated transfer of cholesterol and phospholipids to form HDL-like particles [68, 69] (Fig. 1). Loss of ABCA1 in mouse AD model markedly reduces APOE levels and yields smaller, poorly lipidated APOE-containing particles, indicating that APOE secretion and lipidation are functionally coupled [70]. This pathway is transcriptionally regulated by the Liver X receptor / Retinoid X receptor (LXR/RXR) signaling axis, in which sterol-derived oxysterols activate LXR/RXR heterodimers to induce the expression of genes involved in cholesterol efflux and lipoprotein formation, including APOE and ABCA1, which couples cellular sterol sensing to APOE expression and lipidation [71, 72].

Astrocyte-centered cholesterol pathway is critical for neuronal development and synaptic function. Astrocyte-derived cholesterol promotes synaptogenesis, whereas loss of astrocyte cholesterol synthesis disrupts neurite outgrowth and neuronal function [17, 57, 73, 74]. In parallel, astrocytes contribute to synapse formation and remodeling by coupling lipid supply with the secretion of synaptogenic factors, including thrombospondins (particularly TSP1 and TSP2), hevin/SPARCL1, SPARC, and glypicans, to regulate synaptic development and maintenance [5, 75]. Astrocyte-derived cholesterol also shapes neuronal membrane organization. In neurons, APOE-associated cholesterol delivery regulates lipid raft composition and can influence the localization of amyloid precursor protein (APP), a transmembrane protein that can undergo either non-amyloidogenic or amyloidogenic processing pathways [76]. Under physiological conditions, APP cleavage by α-secretase generates soluble APP-α (sAPP-α), which supports neuronal excitability and synaptic function while preventing amyloid-β (Aβ) formation [77]. In contrast, sequential cleavage by β-secretase and γ-secretases generates Aβ peptides capable of aggregating into amyloid plaques [78]. Because β- and γ-secretases preferentially localize to cholesterol-rich lipid rafts in membrane domains, astrocyte-derived APOE-cholesterol transport can modulate APP trafficking and membrane partitioning, thereby linking a physiological astrocyte lipid pathway to pathological protein generation [79]. A recent study further demonstrates that APOE isoforms differentially regulate neuronal lipid export and intercellular lipid trafficking, including the transport of oxidized unsaturated lipids through ATP-binding cassette transporter A7 (ABCA7), with APOE4 reducing lipid transport efficiency and elevating neuronal ferroptosis and excitotoxicity relative to APOE2 and APOE3 Christchurch (APOE3Ch) [80]. This could represent another isoform-dependent contribution of APOE to AD-related lipid dysregulation and neurodegeneration.

Astrocytic cholesterol metabolism is also regulated by a network of intercellular signaling cues, primarily from neurons and microglia. Neuron-derived 24 S-hydroxycholesterol (24 S-HC), a major product of neuronal cholesterol turnover, activates astrocytic LXRs and promotes the expression of APOE and cholesterol efflux machinery, thereby coupling neuronal sterol demand to astrocytic lipid supply [81]. Beyond neurons, recent work showed that the microglial oxysterol 25-hydroxycholesterol (25-HC) remodels astrocytic lipid metabolism by suppressing sterol synthesis while enhancing cholesterol esterification and the LD accumulation, and extracellular APOE particle secretion [82] (Fig. 1).

Together, these findings highlight that cholesterol synthesis, APOE lipidation, and efflux do not operate as isolated, astrocyte-intrinsic pathways, but rather form a tightly coupled system that is dynamically tuned by neuron-glia and glia-glia communications. Because this system underlies core neuronal function and broader tissue homeostasis, even subtle disturbances in sterol production, APOE particle assembly, or cholesterol trafficking could have consequences that extend beyond lipid balance alone, especially in the long aging process.

### Neuron-to-astrocyte FA transport, storage, and degradation

Neurons are intrinsically vulnerable to lipid stress because they have limited FA oxidation activity under basal conditions as well as low antioxidant capacity, with astrocyte-mediated lipid catabolism especially important during sustained activity or oxidative challenges [24, 83]. Consistent with this unique metabolic capability, astrocytes are enriched in pathways supporting FA uptake, storage, and catabolism, which positions them as key regulators of FA homeostasis in the brain [84] (Fig. 1). Notably, recent studies indicate that under certain metabolic conditions, such as low glucose or high energetic demand, neurons can mobilize triglyceride-derived FAs and use them as an alternative fuel source [85–88]. These observations refine the traditional view by suggesting that neuronal FA metabolism is restricted and context-dependent, whereas astrocytes remain the principal cells specialized for the uptake, storage, detoxification, and catabolism of excess lipid species in the brain.

Under conditions of increased neuronal activity or oxidative stress, neurons generate excess and peroxidized lipid species that are exported to neighboring astrocytes primarily in APOE-associated particles [24, 26, 27]. This pathway functions as an important protective mechanism that relieves neurons from lipotoxic burden. Although neurons can transiently sequester excess lipids into LDs as triglycerides, their intrinsic buffering capacity is limited and becomes insufficient under sustained stress [23, 24, 26, 27].

Once internalized by astrocytes, neuron-derived FAs can be esterified and stored in LDs, which sequester potentially toxic lipid species and limit oxidative damage [24, 26, 27, 89]. Astrocytes then couple LD storage to mitochondrial FA degradation, supported by a transcriptional program inducing genes for lipid catabolism and FA entry into the mitochondria and β-oxidation [23, 90] (Fig. 1). This integration of uptake, storage, and oxidation enables astrocytes to detoxify peroxidized lipids to protect neighboring neurons while partially supporting their own bioenergetic needs [22–24].

Physiologically, the astrocytic FA buffering system is dynamically regulated. Sustained neuronal activity promotes lipid transfer and LD accumulation in astrocytes, whereas the sleep/wake cycle facilitates lipid transport and clearance, and metabolic recovery, highlighting a tightly controlled neuron-glia cycle of FA flux, detoxification and utilization [24, 91]. Astrocytic FA degradation plays a fundamental role in maintaining brain lipid homeostasis. When astrocytic FA oxidation capacity is exceeded, accumulated lipids are redirected into maladaptive signaling pathways. Also, excessive acetyl-CoA, intermediate FA degradation, can be exported to the cytosol to trigger STAT3 acetylation and drive a reactive, pro-inflammatory astrocyte phenotype [22]. At the same time, failure of astrocytic lipid clearance forces neurons to engage in FAO as a compensatory mechanism. Due to neurons’ limited antioxidative capacity, this results in excessive lipid peroxidation, oxidative stress, mitochondrial damage, and synaptic dysfunction [22]. Collectively, these lines of evidence support a physiological framework in which astrocytes serve as the major buffering and degradative compartment for neuronal lipid damage and stress; such dynamic lipid exchange between neurons and astrocytes helps preserve the redox balance and metabolic homeostasis within the brain.

### Astrocytes in myelin lipid supply

**Fig. 2:**
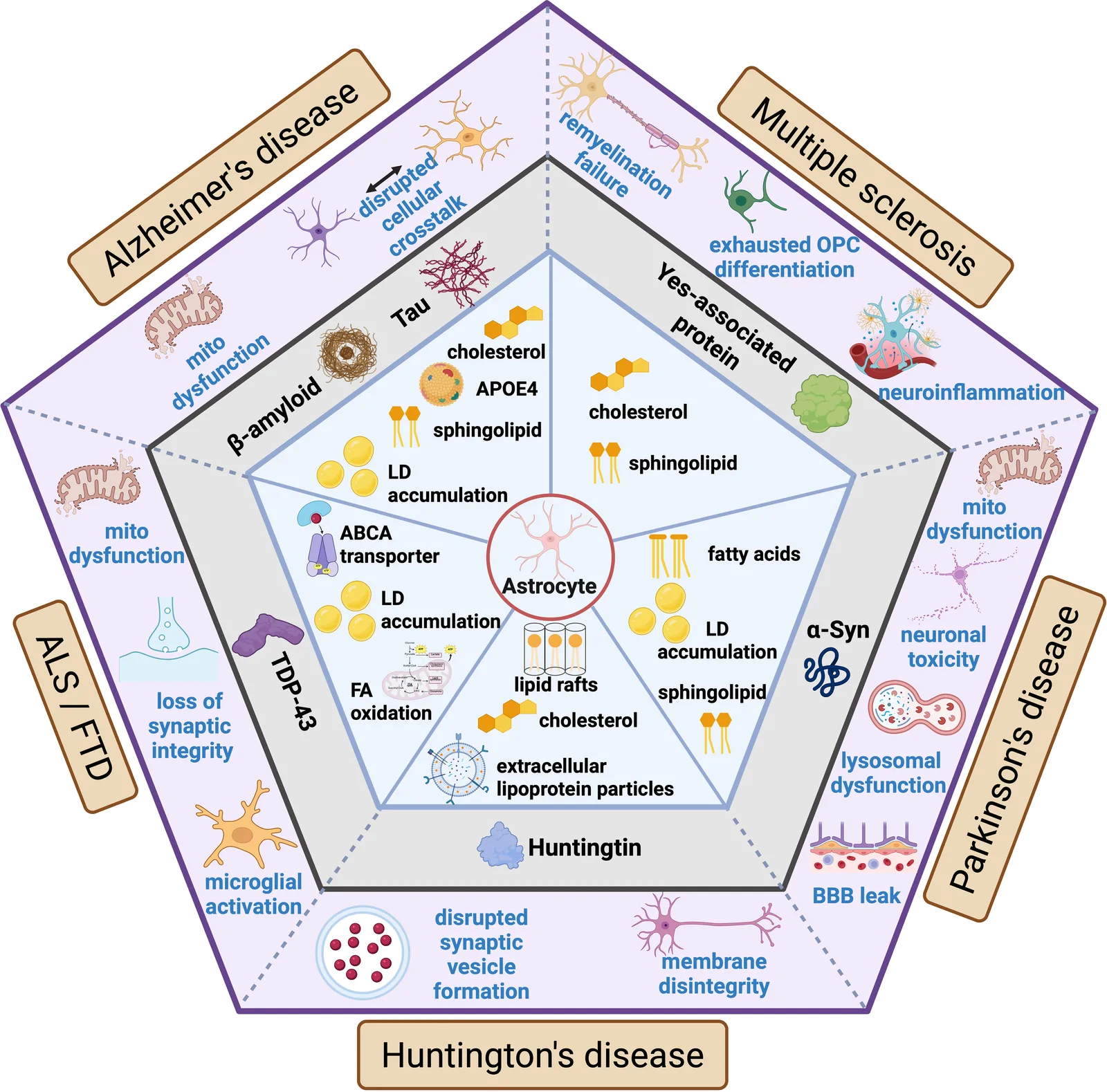
Disease-related components of astrocytic lipid dysregulation. Center circle: astrocyte; Blue pentagram: lipid-related mechanisms in each neurodegeneration disease; Grey pentagram: disease-related proteins including β-amyloid and Tau, Yes-associated protein (YAP), α-synuclein, huntingtin, and TDP-43; Purple pentagram: functional deficits of astrocytic lipid dysregulation in diseases

Astrocytic lipid metabolism is also central to white matter homeostasis. Myelin is an unusually lipid-rich membrane, and its formation and maintenance impose a substantial biosynthetic burden on oligodendrocytes. However, oligodendrocytes do not meet this demand autonomously. Evidence from genetic animal models indicates that CNS myelination depends on both astrocyte-derived lipids and oligodendrocyte-intrinsic FA synthesis, suggesting that myelin formation and repair rely on complementary lipid supply pathways within white matter [21, 92, 93] (Fig. 1). While astrocytic sterol metabolism is involved in myelin maintenance and remyelination, direct evidence for astrocyte-to-oligodendrocyte lipid transfer is limited. Nevertheless, when astrocytic lipid catabolism is disrupted and lipid species accumulate, the *de novo* synthesis of cholesterol, FA and phospholipids are adaptively inhibited in astrocytes, which maladaptively triggers severe demyelination due to compromised replenishment of myelin lipids [22]. In the Experimental Autoimmune Encephalomyelitis (EAE) model of multiple sclerosis, astrocytic Yes-associated protein (YAP) promotes expression of cholesterol synthesis genes including *Hmgcs1*, whereas astrocyte-specific YAP deletion worsens inflammatory infiltration and demyelination. Pharmacological activation of YAP or upregulation of cholesterol synthesis genes partially rescues these deficits [92]. These data support the view that astrocytic lipid metabolism is not only permissive for myelin repair but can actively determine remyelination efficiency and implicated in demyelinating diseases such as multiple sclerosis (Fig. 2).

Together, these findings indicate that astrocyte-dependent lipid supply shapes neuronal and circuit function not only through membrane support and metabolic buffering, but also through maintenance of myelin integrity and axonal resilience. Disruption of this astrocytic support may therefore promote neurodegeneration by compromising both neuron-glia metabolic coupling and the lipid resources needed for white matter maintenance and repair.

## Pathological remodeling of astrocytic lipid metabolism in neurodegeneration

In neurodegenerative disorders, the transition of astrocytes from homeostatic supporters to pathogenic drivers is associated, and potentially mediated, by a failure in specific molecular and metabolic programs that integrate lipid sensing, metabolic flux, lipid clearance, and inflammatory signaling.

### Cholesterol dyshomeostasis and APOE-dependent remodeling of astrocyte lipid profile

The decoupling of astrocytic cholesterol synthesis from effective sterol export and neuronal delivery is a core pathological event of lipid remodeling, largely connected with APOE polymorphism. The APOE ε4 allele (APOE4) is not only the strongest genetic risk factor for late-onset Alzheimer’s disease (AD) [1], but also a risk modifier for other neurodegenerative diseases [2, 3]. Functionally, human APOE isoforms differ in lipidation efficiency and lipoprotein particle properties. In the CNS, APOE4 is generally less efficiently lipidated and is associated with smaller, less stable APOE-containing HDL-like particles than APOE3 or APOE2, differences that are likely to influence lipid transport efficiency and neuronal lipid homeostasis [25, 68, 94]. In human induced pluripotent stem cell (iPSC)-derived models and postmortem tissues, APOE4 drives astrocytic lipid dysregulation characterized by increased *de novo* cholesterol synthesis despite elevated intracellular cholesterol, in part because of lysosomal cholesterol sequestration and impaired handling of sterol pools [25, 95] (Fig. 1). Mechanistically, recent work also suggests that APOE4 exhibits increased interaction with low-density lipoprotein receptor (LDLR)-associated trafficking pathways, which enhances cholesterol uptake but impairs lysosomal cholesterol recycling and efflux, thereby promoting intracellular cholesterol trapping and lysosomal lipid accumulation [96].

Beyond these effects on secreted lipoprotein-mediated cholesterol trafficking, APOE also regulates intracellular lipid metabolism in a cell-autonomous manner. Recent work shows that APOE traffics to the surface of astrocyte LDs, where it modulates droplet size and triglyceride saturation, with APOE4 promoting larger, more unsaturated, and more peroxidation-sensitive LDs [89]. APOE4 alters astrocytic mitochondrial morphology toward fragmentation, which is associated with reduced FA β-oxidation capacity [23]. APOE has also been shown to undergo isoform-dependent trafficking from post-Golgi compartments to autophagic and lysosomal pathways [97]. Together, these findings suggest that APOE isoforms differentially regulate both extracellular lipid transport and intracellular lipid processing, which may contribute to the complexity and context dependence of APOE isoform-associated protective and pathogenic mechanisms in neurodegenerative diseases.

Consistent with these mechanisms, high-resolution lipidomics from the Neurolipid Atlas demonstrates that APOE4 drives a massive intracellular accumulation of cholesteryl esters in astrocytes, a signature that mirrors lipid profiles in human AD brains [98]. In tauopathy models, APOE4 also promotes glial lipid accumulation and perturbations in cholesterol metabolism and lysosomal function, whereas increasing glial efflux through LXR agonism or ABCA1 overexpression attenuates tau pathology and neurodegeneration [99]. In AD, disturbed astrocytic cholesterol handling has direct consequences for neuronal membrane organization and amyloidogenic signaling. Astrocyte-derived cholesterol regulates neuronal membrane nanostructure, APP trafficking, and amyloidogenic APP processing through modulation of lipid raft organization, while experimental suppression of astrocytic cholesterol synthesis has been shown to decrease Aβ burden and tau-associated pathology in the 3xTG AD mouse model [94]. The same mechanism is implicated beyond AD: in Huntington’s disease, astrocytes carrying mutant huntingtin secrete less APOE-lipoprotein-bound cholesterol, and impaired astrocyte-neuron cholesterol crosstalk directly compromises neurite outgrowth and synaptic support [100] (Fig. 2).

Moreover, studies from genetic models of APOE manipulation and protective APOE isoforms suggest that APOE-associated lipid abnormalities are at least partially modifiable. In particular, APOE2 is associated with distinct lipid-enriched metabolic profiles in vivo, consistent with a protective lipid program [101]. APOE4-to-APOE2 switching in mice reverses AD-relevant lipidomic and transcriptomic changes, particularly in astrocytes, and improves cognition and amyloid pathology in the 5xFAD mouse model of AD [102, 103]. Astrocyte-specific manipulation is especially informative. Astrocyte-restricted APOE4-to-APOE2 switching is sufficient to reduce Aβ pathology, decrease gliosis, and improve cognition [102]. Consistently, selective removal of astrocytic APOE4 decreased tau pathology, as well as tauopathy-induced synaptic loss and microglial phagocytosis of synapses [104]. The APOE-R136S Christchurch variant protects against APOE4- or familial AD-driven amyloid and tau pathology, neuroinflammation and neurodegeneration in mice [105–107]. In line with its neuroprotective effect, the APOE3-Christchurch human astrocytes display a distinct lipidomic profile, including reduced ceramides and lower gamma-linolenic acid, consistent with a less pro-inflammatory status [108].

### Failure of lipid disposal: LDs, FAO, and reactivity

A second major point of pathological lipid-related remodeling is the breakdown of neuron-astrocyte coupling of FA metabolism, at the levels of intercellular trafficking, LD storage, and mitochondrial degradation (Fig. 2). Astrocytic mitochondrial dysfunction and reduced FA oxidation capacity are sufficient to trigger progressive LD accumulation, astrocyte reactivity and AD-resembling neuroinflammation and neurodegeneration [22]. Mechanistically, FA overload or impaired mitochondrial oxidative phosphorylation elevates intracellular acetyl-CoA levels and promotes the acetylation and activation of STAT3, the key transcriptional regulator of astrocyte reactivity, which establishes a direct connection between defective lipid catabolism and reactive astrocyte states [22]. In AD-related models, both amyloidosis and APOE4 can trigger similar disruption to astrocytic FA degradation and LD accumulation [22, 23]. In these contexts, LD accumulation may initially function as a mechanism for lipid storage and buffering against lipotoxicity, but when the lipid burden overwhelms astrocytic catabolic and storage capacity, LDs may represent a signal that astrocytes can no longer efficiently and timely process incoming lipid load. Consistent with this notion, excessive astrocytic LD accumulation leads to the formation of a pathological astrocyte state associated with mitochondrial oxidative phosphorylation deficits in human temporal lobe epilepsy and mouse epilepsy models, which promotes neuronal hyperactivity, neuroinflammation and degenerative remodeling [109]. Moreover, in the context of amyotrophic lateral sclerosis (ALS) and frontotemporal degeneration (FTD), astrocytes with TDP-43 inclusions accumulate substantially more LDs than wild-type astrocytes [110]. Astrocytes carrying the C9orf72 expansion (hexanucleotide repeat expansion in chromosome 9 open reading frame 72), the most common genetic cause of familial ALS and FTD [111], show reduced metabolic flexibility and increased starvation-induced toxicity, consistent with a broader failure of astrocytic stress adaptation, although the lipid-specific astrocyte mechanisms remain less understood than in AD [112]. In PD models, patient-derived astrocytes display altered metabolism, calcium handling, cytokine release, and lipid metabolite changes, supporting the idea that impaired astrocytic metabolic flexibility may also contribute to, or be reprogrammed by, synucleinopathy [113].

These findings suggest that excessive LD accumulation is not merely a disease-specific byproduct but reflects broader astrocytic metabolic shift and impaired lipid disposal capacity. Across AD, ALS/FTD, PD, and epilepsy-related conditions, pathological astrocytes appear to converge on a shared state characterized by impaired lipid catabolism, mitochondrial stress, inflammatory reprogramming, and reduced ability to support neuronal homeostasis.

### Lipid-derived inflammatory signaling

Beyond defects in lipid storage and degradation, pathological astrocytes also undergo inflammatory lipid reprogramming in which sterol, phospholipid, and sphingolipid metabolites become active signaling mediators that trigger or amplify glial dysfunction and neurodegeneration. In inflammatory settings, activated microglia produce 25-HC, which astrocytes take up and respond to by increasing ABCA1-dependent efflux, suppressing SREBP2-driven synthesis, and storing more cholesteryl esters in LDs [82]. Notably, 25-HC elevates extracellular APOE more effectively in APOE3 than APOE4 astrocytes [82]. This difference is functionally relevant because APOE3-containing lipoprotein particles are more effectively lipidated than APOE4 particles in supporting cholesterol redistribution and neuronal lipid homeostasis. By contrast, the reduced ability of APOE4 astrocytes to generate functionally effective extracellular APOE may further limit sterol delivery and lipid buffering under conditions of inflammatory stress. Although 25-HC can activate sterol-responsive pathways and cholesterol efflux, sustained 25-HC signaling occurs in a pro-inflammatory setting and may become maladaptive by exacerbating lipid dysregulation and inflammatory activation.

In AD, inflammatory lipid signaling in astrocytes is also shaped by cytoplasmic phospholipase A2 (cPLA2), which releases arachidonic acid from membrane phospholipids and triggers downstream pro-inflammatory signaling [114]. APOE4 increases cPLA2 phosphorylation and activity and pro-inflammatory signaling in astrocytes [115]. More recent work further connects cPLA2 activation to AD-related synaptic injury [116]. Peripheral-to-central sterol signaling also plays a role. 27-HC can cross the blood–brain barrier and promotes α-synuclein aggregation, seeding, and spread in PD models [117] (Fig. 2).

Importantly, reactive astrocytes not only fail to clear toxic lipids, but can also actively generate and secrete neurotoxic lipid species. Inflammatory astrocytes were shown to release *de novo* synthesized long-chain saturated FAs that impair neuronal and oligodendrocyte survival, suggesting that pathological astrocytic lipid remodeling also involves a shift from protective lipid buffering toward pathogenic lipid secretion [118].

Sphingolipid and glycolipid programs could also become pathogenic in chronic CNS inflammation. In PD, glucosylceramidase beta 1 (GBA1) mutation-associated loss of glucocerebrosidase activity promotes lysosomal lipid abnormalities, autophagy defects, and impaired α-synuclein clearance; although this finding is not astrocyte-exclusive, it is consistent with the view that astrocytic lysosomal sphingolipid dysfunction can contribute to synucleinopathy progression [119, 120]. In EAE and potentially MS, astrocytic sphingolipid metabolism drives a cPLA2-driven, NFκB- and hexokinase-mediated mechanism that promotes inflammation and reduces neuron-supportive lactate production, while pharmacologic suppression of the upstream lipid signaling by Miglustat ameliorates disease progression [121]. Although the specific lipid species differ across diseases, these studies collectively support a convergent model in which pathological lipid changes are not only markers of metabolic dysfunction but also function as active drivers of astrocyte inflammatory reprogramming and neuronal injury. Thus, oxysterols and sphingolipids could represent links between lipid dyshomeostasis and inflammatory astrocyte activity, with AD and PD better exemplifying sterol-derived signals and multiple sclerosis better exemplifying glycolipid-driven pathogenic astrocytes. These findings suggest that in addition to their metabolic roles, perturbations to astrocytic lipid metabolism can also directly contribute to neuroinflammation through lipid-derived cascades.

## Therapeutic strategies targeting astrocytic lipid metabolism

### Restoring APOE-mediated cholesterol efflux

The most studied lipid-correcting therapeutic strategy against neurodegeneration is restoration of cholesterol efflux and APOE lipidation through the LXR/RXR-ABCA1-APOE pathway. By enhancing the efficiency of cholesterol efflux and the maturation of APOE-containing lipoproteins, this strategy aims to alleviate intracellular lipid accumulation while restoring essential metabolic support to neurons, especially in APOE4-related settings where APOE particles are relatively poorly lipidated. LXR and RXR can form heterodimers in the brain and function as central sensors of lipids including cholesterol and oxysterols [122]. In preclinical models, RXR activation by Bexarotene increased ABCA1, ABCG1, and APOE expression, enhances APOE lipidation and secretion of HDL-like lipoprotein particles, and reduces soluble Aβ burden [123–127]. In APOE4 astrocytes, which are characterized by lysosomal cholesterol sequestration, impaired cholesterol trafficking, and poorly lipidated APOE particles, ABCA1 activation bypasses these trafficking defects and improves lipid efflux capacity. Restoration of the lipidome reduces the metabolic stress that typically drives these cells toward a neurotoxic reactive state [25, 82]. However, clinical findings with this strategy have been mixed. Bexarotene treatment in humans showed poor brain penetration, produced only a modest increase in cerebrospinal fluid (CSF) APOE, and did not alter Aβ kinetics [128]. More recent work nevertheless supports the biological validity of this approach: in APOE4/tauopathy models, improving glial lipid efflux with an LXR agonist reduced glial lipid accumulation and neurodegeneration [99]. A practical takeaway is that therapeutic enhancement of APOE lipidation and cholesterol efflux remains biologically promising, although future development will likely need better brain-penetrant and better tolerated modulators than first-generation LXR/RXR agonists.

### Preserving astrocytic FA turnover and mitochondrial function

**Fig. 3:**
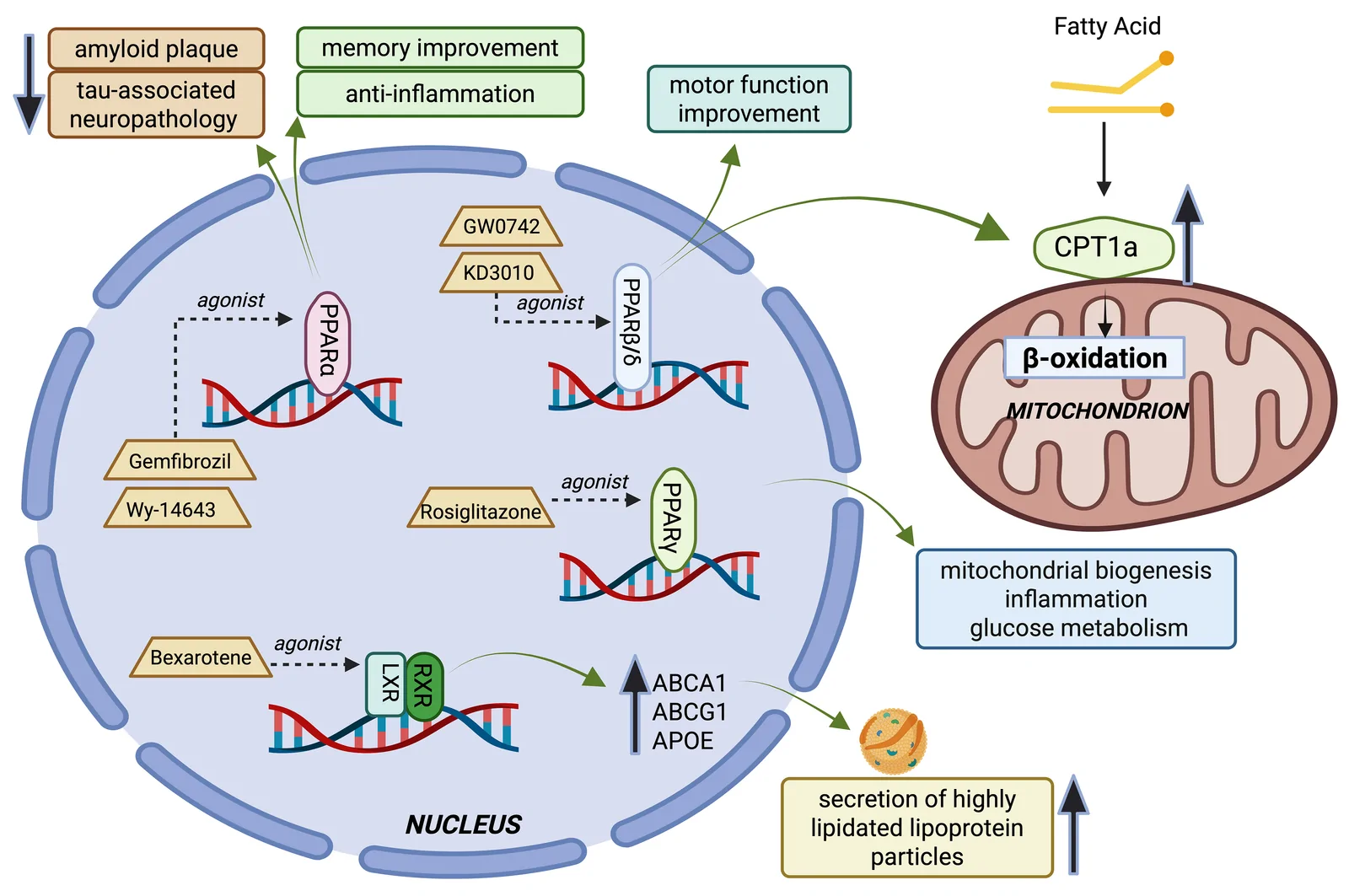
Therapeutic strategies targeting astrocytic lipid metabolism via PPAR nuclear receptors. PPAR nuclear receptors regulate astrocytic lipid handling and are key targets for neurodegeneration diseases. RXR agonist Bexarotene activates LXR/RXR complex to increase expression of ABCA1, ABCG1 and APOE, and enhances secretion of highly lipidated lipoprotein particles. PPARβ/δ agonist GW0742 rescued defective FA oxidation by increasing CPT1A expression and promoting mitochondrial β-oxidation. PPARδ agonist KD3010 improves motor function. PPARγ agonism links with mitochondrial biogenesis, inflammation, and glucose metabolism. PPARα agonism alleviates amyloid plaque burden and tau-associated neuropathology, improves memory and exhibit anti-inflammatory effects

A second strategy is to preserve astrocytic handling of FAs by improving mitochondrial competence and FA oxidation. Peroxisome proliferator-activated receptors (PPARs) are a family of nuclear receptors regulating energy and lipid metabolism (Fig. 3). The three known PPAR isoforms are α, γ and δ (also known as β), each of which has different roles, affinities, and distribution throughout the body [129]. PPARγ enhances insulin sensitization and glucose metabolism. PPARα is involved in FA oxidation and has a potential role in redox regulation, whereas PPARβ/δ can also facilitate FA metabolism and plays a role in insulin signaling [130, 131]. Thus, therapeutic enhancement of PPAR activity to promote astrocytic mitochondrial FA turnover and metabolic flexibility may represent a strategy to mitigate lipid accumulation, inflammatory reprogramming, and loss of neuronal support across different neurodegenerative diseases.

An established astrocyte-linked study is PPARβ/δ agonism. In human iPSC-derived AD astrocytes, the PPARβ/δ agonist GW0742 rescued defective FA oxidation by increasing the expression of CPT1A, a rate-limiting enzyme for mitochondrial FA import and oxidation. In the same study, GW0742 treatment in APP/PS1 mice increased CPT1A expression and improved memory in a fear-conditioning task [132]. GW0742 was also found capable of reducing loss of dopaminergic neurons in 1-methyl-4-phenyl-1,2,3,6-tetrahydropyridine (MPTP)-induced mouse model of PD [133]. In HD model, PPARδ agonist KD3010 improved motor function, reduced neurodegeneration, and increased survival [134] (Fig. 3).

The broader PPAR-directed approaches are also of interest, but the translational potential is less understood, and the degree of astrocyte specificity is often unclear. PPARγ agonists generated substantial interest in AD because of their links to mitochondrial biogenesis, inflammation, and glucose metabolism [135], but it remains unclear whether and how PPARγ influences lipid metabolism in astrocytes. Rosiglitazone, a PPARγ agonist, generated early interest, including subgroup signals in APOE4-negative participants [136], but phase III studies in mild-to-moderate AD yielded overall negative outcome [136, 137].

In the brain, PPARα is predominantly expressed in astrocytes [138, 139], although the therapeutic effects of astrocytic PPARα activation merit further evaluation. In APP/PS1 mice, PPARα agonist gemfibrozil reduced amyloid plaque burden and improved memory in a PPARα-dependent manner, and more recent work showed that gemfibrozil lowered tau-associated neuropathology in a MAPT mouse model [140, 141]. Another PPARα agonist Wy-14,643 also alleviates AD-related pathologies and cognitive deficits in the APP/PS1 mice [142, 143]. In a model of ALS, PPARα agonists exhibit anti-inflammatory and pro-survival effects [144]. In the MPTP model of PD, gemfibrozil also protected dopaminergic neurons through a PPARα-dependent astrocytic GDNF pathway, which is mechanistically interesting even though it is not a direct lipid-mediated pathway [145]. These data suggest that PPARα activation can engage astrocyte-related trophic and metabolic pathways, although its lipid-metabolic effects remain less directly resolved.

Together, these findings support a model in which preservation of astrocytic mitochondrial competence and FA turnover may mitigate multiple downstream pathological processes, including LD accumulation, inflammatory reactivity, oxidative stress, and loss of neuronal metabolic support. Future astrocyte-specific studies with lipid or mitochondrial characterizations will be important for determining whether these benefits reflect direct correction of astrocytic lipid metabolism or broader systems-level effects (Fig. 3).

### Targeting pathogenic lipid signaling

A complementary therapeutic strategy is to target pathogenic lipid signaling rather than directly altering lipid levels and distribution. This approach is particularly relevant when astrocytic lipid remodeling has shifted the cellular status toward inflammatory state. Two strategies focusing on the metabolism of the pro-inflammatory arachidonic acid will be discussed including monoacylglycerol lipase (MAGL) and cPLA2.

**Fig. 4:**
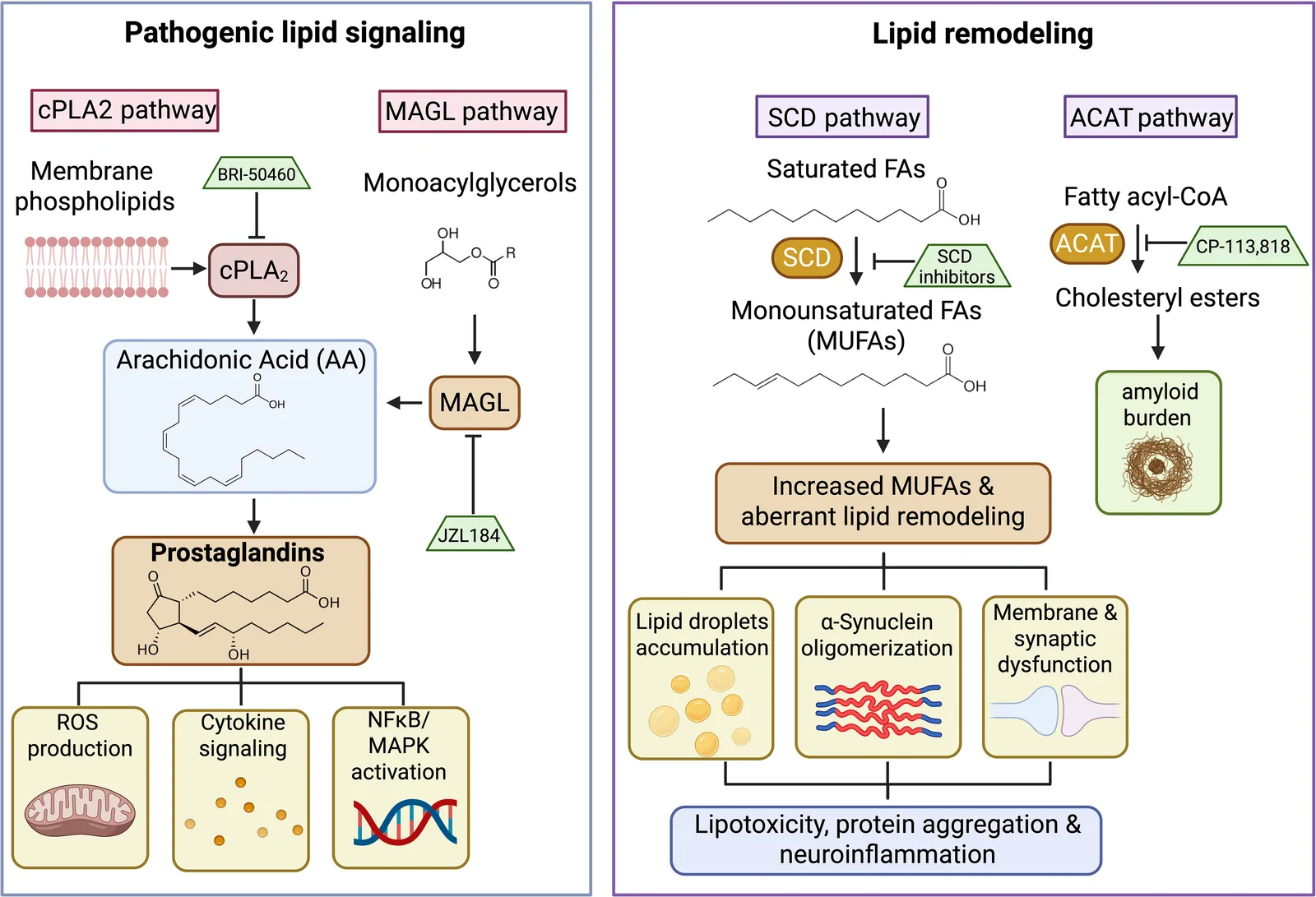
Therapeutic targets in pathogenic lipid signaling and lipid remodeling pathways. Left: Pathological conditions activate the cPLA2 and MAGL pathways to generate inflammatory arachidonic acid (AA), which is metabolized into prostaglandins that drive downstream mitochondrial ROS production, cytokine signaling, and NFκB/MAPK activation. This pathogenic signaling can be suppressed by cPLA2 inhibitor BRI-50,460 or the MAGL inhibitor JZL184. Right: Lipid remodeling by SCD converts saturated FAs into monounsaturated FAs (MUFAs); accumulated MUFAs trigger LD accumulation, α-synuclein oligomerization, and synaptic dysfunction, leading to lipotoxicity and neuroinflammation, which can be mitigated by SCD inhibitors. The ACAT pathway converts fatty acyl-CoA and cholesterol into neutral cholesteryl esters, a process that can be targeted by the inhibitor CP-113,818 to disrupt cholesterol esterification and reduce amyloid plaques

MAGL is a key regulator of brain arachidonic acid metabolism and inflammatory lipid signaling through its hydrolysis of the endocannabinoid 2-arachidonoylglycerol (2-AG), which releases free arachidonic acid as a precursor for pro-inflammatory eicosanoids [146]. As excessive arachidonic acid-derived signaling contributes to chronic glial activation and neurodegeneration in AD, including through COX-2-dependent prostaglandin pathways and broader oxidative lipid cascades, MAGL inhibition has emerged as a potential therapeutic strategy that simultaneously reduces arachidonic acid release from monoacylglycerols while increasing endocannabinoid tone to dampen downstream inflammatory lipid signaling [147–149]. In AD mouse models, MAGL inhibitor JZL184 reduced inflammatory responses in both astrocytes and microglia, supporting a glia-relevant anti-inflammatory effect even though the in vivo benefit may not be astrocyte-exclusive [150, 151]. In a chronic MPTP model of PD, JZL184 was also neuroprotective by preserving dopaminergic neurons and suppressing inflammatory glial phenotypes, suggesting that modulation of lipid-derived inflammatory signaling can improve disease-relevant outcomes across diseases [152] (Fig. 4).

As discussed above, in pathogenic astrocytes, cPLA2 links membrane phospholipid remodeling to the production of arachidonic acid, and couples lipid metabolism to inflammatory programs. Studies in AD, EAE and related models identified cPLA2 as a mechanistically central regulator of lipid-driven astrocyte pathogenicity [115, 121]. Translationally, brain-penetrant and selective cPLA2 inhibitors are being actively developed for AD-relevant neuroinflammation. The lead compound BRI-50,460 showed favorable CNS penetrance, and in human iPSC-derived astrocytes and neurons, it reduced Aβ oligomer-induced cPLA2 activation, tau hyperphosphorylation, and synaptic loss [153]. Although the cellular effects in vivo remain to be determined, these findings make cPLA2 one of the promising examples of a lipid-inflammatory pathway with both astrocytic relevance and translational potential (Fig. 4).

### Other lipid metabolic targets

Several additional lipid-modifying strategies have emerged and been tested in animal models. Among these, stearoyl-CoA desaturase (SCD) inhibition is a promising candidate. SCD regulates FA saturation by catalyzing the conversion of saturated FAs to monounsaturated FAs [154]. SCD levels are correlated with triglyceride levels but are also induced by dietary carbohydrates and cholesterol through activation of SREBP-1c and LXR [155]. In the 3xTg AD mouse model, short-term brain SCD inhibition reversed immune, synaptic, and cognitive impairments, indicating that altering FA desaturation can mitigate disease-relevant lipid, inflammatory, and neuronal impairments [156]. In parallel, SCD inhibition has shown efficacy in synucleinopathy models. Pharmacologic SCD inhibition rescued α-synuclein toxicity in neural cells and prevented multiple Parkinson-related phenotypes in α-synuclein transgenic mice [157, 158]. Although these studies are not astrocyte-specific in vivo, they remain relevant as astrocytes are major regulators of brain FA metabolism (Fig. 4).

Another emerging strategy is the modulation of cholesteryl ester metabolism. Acyl-coenzyme A: cholesterol acyltransferases (ACATs) are membrane-bound proteins that convert long-chain fatty acyl-CoA and cholesterol to cholesteryl esters [159]. ACAT inhibitor CP-113,818 markedly reduced amyloid plaque burden, insoluble Aβ, and brain cholesteryl esters in an amyloidosis mouse model, suggesting that reducing the neutral esterified cholesterol storage can influence amyloidogenic processing and plaque accumulation [160, 161]. However, the mechanistic contribution of astrocytes to these effects remains less understood than microglial mechanisms [48]. There is also accumulating mechanistic evidence supporting triglyceride and LD metabolic pathways as targets for neurodegeneration, such as diglyceride acyltransferase (DGAT), LD-associated perilipins (PLINs), and adipose triglyceride lipase (ATGL), although direct therapeutic validation and connection to astrocytic lipid metabolism remain limited [46, 162–164] (Fig. 4).

### Challenges in modeling and targeting astrocytic lipid metabolism

Despite the progress, several limitations remain in the current understanding and therapeutic targeting of astrocytic lipid metabolism in neurodegeneration. Much of the mechanistic evidence derives from rodent models, whereas human astrocytes exhibit distinct morphological, transcriptional, and metabolic characteristics that may influence lipid handling and inflammatory responses [2, 165, 166]. Several APOE4-associated phenotypes, including lysosomal cholesterol sequestration, impaired lipid efflux, and cholesteryl ester accumulation, are robustly observed in human iPSC-derived astrocytes and supported by human tissue-linked analyses, underscoring the importance of human model systems for resolving astrocytic lipid pathology [25, 95, 98]. Although iPSC-derived systems provide important human-relevant platforms, they do not fully recapitulate the aged, multicellular, and long-duration tissue context of the adult human brain. In parallel, current lipidomic and metabolomic approaches still have limited ability to resolve cell type-specific lipid flux, spatial heterogeneity, and the functional distinction between adaptive lipid remodeling and pathogenic lipid accumulation in vivo. These challenges are compounded by the marked heterogeneity of reactive astrocyte states across brain regions, disease stages, and neurodegenerative conditions [9]. Future studies integrating single-cell and spatial transcriptomics with lipidomics, metabolomics, and functional imaging in rodent models, human tissues and patient-derived models will be important for defining disease-relevant and cell type-resolved lipid states and identifying therapeutically actionable metabolic pathways.

## Conclusions and perspectives

Astrocytic lipid metabolism is essential for brain homeostasis and has emerged as a point of convergence across neurodegenerative pathologies. In the healthy brain, astrocytes coordinate cholesterol synthesis and distribution, FA buffering and clearance, and multiple forms of lipid-mediated communications with neighboring cells. These functions are integral to membrane integrity, synaptic function, myelin turnover, and adaptation to metabolic and inflammatory stress. In neurodegenerative diseases, however, the same pathways can be progressively rewired into maladaptive programs that trigger or sustain astrocyte reactivity and inflammation, exacerbate metabolic deficits and proteinopathies, compromise neuronal support, and ultimately contribute to neuronal loss.

A major open question moving forward is determining when astrocytic lipid responses remain adaptive and when they become pathogenic. Astrocytes normally mitigate neuronal lipid stress through uptake, storage, and catabolism of excess FA, yet under chronic stress conditions, these protective responses can become inefficient, unresolved, or exhausted. For example, it remains to be determined when and how astrocytic LDs, which are initially protective lipid buffers, become overwhelmed and transition into pathogenic states. More broadly, astrocytic lipid biology is increasingly recognized as context-dependent, varying across disease stage, regional environment, neuronal activity, and across states such as sleep-wake and circadian cycles. Understanding this temporal and functional plasticity will be essential for defining what aspects of these pathways are upstream drivers, what are targetable and reversible, and when is the optimal intervention window.

As the field now rapidly moving beyond descriptive snapshots of lipid alterations towards a more mechanistic understanding of lipid metabolic machinery and lipid signaling, the key questions are no longer the lipid species changes in astrocytes and the brain, but which lipid programs preserve metabolic homeostasis and neuronal support, which mark transition into vulnerability, and which actively drive degeneration. It is also important to determine which lipid alterations are shared across neurodegenerative diseases and which are proteinopathy-, cell type-, or region-specific. Resolving these questions will be important to establish astrocytic lipid metabolism not only as a useful mechanistic framework to understand neurodegeneration, but also as a tractable entry point for developing disease-modifying therapeutic interventions.

## Data Availability

No datasets were generated or analysed during the current study.
